# Clonal diversity and genetic profiling of antibiotic resistance among multidrug/carbapenem-resistant *Klebsiella pneumoniae* isolates from a tertiary care hospital in Saudi Arabia

**DOI:** 10.1186/s12879-018-3114-9

**Published:** 2018-05-03

**Authors:** Taher uz Zaman, Maha Alrodayyan, Maha Albladi, Mohammed Aldrees, Mohammed Ismail Siddique, Sameera Aljohani, Hanan H. Balkhy

**Affiliations:** 1 Infectious Diseases Section, King Abdullah International Medical Research Center National Guard Health Affairs, P.O. Box 22490, Mail Code 1515, Riyadh, 11426 Kingdom of Saudi Arabia; 2 King Saud Bin Abdul-Aziz University of Health Sciences, Riyadh, Kingdom of Saudi Arabia; 3 Deccan College of Medical Sciences, Hyderabad, India; 4 Microbiology Section- King Abdul-Aziz Medical City, Riyadh, Kingdom of Saudi Arabia; 5 Infection Prevention and Control Prevention, King Abdul-Aziz Medical City, National Guard Health Affairs, P.O. Box 22490, Riyadh-11426 Riyadh, Kingdom of Saudi Arabia

**Keywords:** Carbapenem-resistant *Klebsiella pneumoniae* (CR*KP*), Clonal diversity, Resistance determinants, Molecular epidemiology, Plasmid profile, Saudi Arabia

## Abstract

**Background:**

The nexus between resistance determinants, plasmid type, and clonality appears to play a crucial role in the dissemination and survival of carbapenem-resistant *Klebsiella pneumoniae* (CR*KP*). The incidence of infections involving CR*KP* in Saudi Arabia is increasing and there is a need for detailed molecular profiling of this pathogen for CR*KP* surveillance and control.

**Methods:**

The resistance determinants of 71 non-redundant CR*KP* isolates were investigated by polymerase chain reaction (PCR) and sequencing. Plasmid typing was performed using PCR-based replicon typing and the clonality of isolates was determined by multilocus sequence typing. Capsular polysaccharide synthesis genes and other virulence factors were examined using multiplex PCR. Diversity was calculated using DIVEIN, clonal relationship was determined using eBURST, and phylogenetic analysis was performed using SplitsTree4.

**Results:**

A polyclonal *OXA-48* gene alone was the most common carbapenemase detected in 48/71 (67.6%) isolates followed by *NDM-1* alone in 9/71 (12.7%) isolates. Coproduction of *OXA-48* and *NDM-1* was observed in 6/71 (8.5%) isolates. Both carbapenemase genes could be transferred into an *Escherichia coli* recipient. *CTX-M-15* was the most abundant extended-spectrum *β-lactamase* gene detected in 47/71 (66.2%) isolates, whereas clone-specific *CTX-M-14* (ST-199 and -709) was found in 15/71 (21%) isolates. Sixty-seven of 71 isolates were positive for one or more plasmid replicons. The replicons detected were: IncFII; IncFIIK; IncFIA; IncFIB; L/M; IncI1; and IncN. FIIK and L/M were predominant, with 69 and 67% positivity, respectively. All isolates were negative for the *magA* (*K1*), *rmpA,* and *K2* genes and presented a non-hypermucoviscous phenotype.

**Conclusion:**

A polyclonal CR*KP* reservoir of sequence types (STs)-37, − 199, and − 152 was observed and ST-152 appeared to be a “frequent carrier” of the *NDM-1* gene. ST-199, a singleton not previously reported, showed a sequence diversity suggestive of positive selection. A significant association was evident between resistance determinants and the clonal types of *K. pneumoniae*: all ST-152 isolates were positive for *NDM-1* but negative for *OXA-48*; ST-199 isolates were positive for *OXA-48* but negative for *NDM-1*; and ST-709 and -199 isolates were positive for *CTX-M-14*. The incidence of certain clonal types in large numbers predicts an outbreak-like situation and warrants stringent surveillance and infection control.

**Electronic supplementary material:**

The online version of this article (10.1186/s12879-018-3114-9) contains supplementary material, which is available to authorized users.

## Background

*Klebsiella pneumoniae* (*KP*), a member of the Enterobacteriaceae family of Gram-negative bacteria, is an important human and animal pathogen [1]. High mortality rates are associated with infection with carbapenemase-producing *KP* [2]. Various carbapenemase genes, such as *KP carbapenemase* (*KP*C), *NDM-1*, *VIM*, *IMP*, and *OXA-48*, have been identified in *KP* [3, 4], some of which appear to be clone-specific and such clones form a reservoir for infection [5]. Although reports are available on the emergence of drug-resistant *KP* in Saudi Arabia [4, 6], few studies have examined the molecular basis of antibiotic resistance, plasmid profiles, virulence factors, and clonal diversity of drug-resistant *KP* in this country. In order to perform adequate infection control interventions and solicit support for effective stewardship programs, local data on antimicrobial resistance mechanisms and the clonal spread are important. Hence, baseline information on resistant clones assists in assessing the trends of resistance as well as their local epidemiology. Therefore, in this study, we investigated the clonal diversity and genetic profile of a population of multidrug-resistant, carbapenem-resistant *KP* (CR*KP*) isolates from patients admitted to our hospital in Riyadh, Saudi Arabia during years 2011 and 2012.

## Methods

### Bacterial isolates and testing of antibiotic resistance

Seventy-one non-redundant multidrug-resistant CR*KP* isolates were obtained from patients attending the Microbiology Section of the King Abdulaziz Medical City, Riyadh, Saudi Arabia, between January 2011 and December 2012. The isolates were identified to the species level using matrix-assisted laser desorption/ionization time-of-flight mass spectrometry (bioMérieux, Marcy-l’Étoile, France). The frozen isolates were sub-cultured on blood agar at 37 °C overnight. Single colonies were picked up and grown in liquid tryptic soy broth(TSB) medium 37 °C in a shaking incubator at 250 rpm overnight. Minimum inhibitory concentrations (MICs) were determined using a Micro VITEK® 2 microbial identification instrument (bioMérieux). The MIC breakpoints for carbapenem were defined according to the modified 2010 Clinical and Laboratory Standards Institute guidelines [7]. Isolates found to have elevated MICs for carbapenem by Micro VITEK® 2 analysis were confirmed by the manual ETEST® (bioMérieux) to have reduced susceptibility to either imipenem or/and meropenem.

### Characterization of resistance genes

Bacterial DNA was isolated using a MagNA Pure kit (Roche Diagnostics, Risch-Rotkreuz, Switzerland). Uniplex polymerase chain reaction (PCR) was performed for *bla*-SHV, *bla*-TEM-1, *bla*-CTX-M, *bla*-*KP*C, *bla*-OXA-48, *bla*-VIM, and *bla*-IMP using primers reported previously [8]. Appropriate positive and negative controls were run simultaneously. Each PCR reaction was conducted at least twice. The primers 5’-GGTTTGGCGATCTGGTTTTC-3′ (F) and 5’-CGGAATGGCTCATCACGATC-3′ (R) were used for the amplification of *bla*-NDM-1, and the primers 5‘-CACCTCATGTTTGAATTCGCC-3′ (F) and 5‘-CTCTCTCACATCGAAATCGC-3′ (R) were used to determine its genetic environment [9]. The PCR products were sequenced on an ABI 3100 DNA analyzer (Life Technologies, Carlsbad, CA, USA). The DNA sequences were analyzed using SeqMan (DNASTAR, Inc., Madison, WI, USA) and MEGA4[10] and blasted against GenBank (National Center for Biotechnology Information, Bethesda, MD, USA). PCR-based replicon typing (PBRT) was performed on these isolates to identify the plasmids as per the protocol described in the PubMLST database (https://pubmlst.org/plasmid/).

### Conjugation experiments

Conjugation experiments were performed on selected isolates (Table 2) using J53Az^r^ *Escherichia coli*, a strain resistant to sodium azide, as a recipient (a kind gift from Dr. Timothy Walsh, University of Cardiff, Wales, UK). Cultures from both donor and recipient bacteria were grown in lysogeny broth (LB) to the logarithmic phase, mixed at a donor:recipient ratio of 1:3, and incubated at 37 °C overnight without shaking. Transconjugants were selected on LB agar plates supplemented with 100 μg/ml of sodium azide and 100 μg/ml of ampicillin. Positive clones were screened by colony PCR for the target resistance genes.

### Detection of hypermucoviscocity phenotyping and virulence associated genes

Bacterial isolates grown on blood agar plates were checked for hypermucoviscocity. Hypermucoviscocity was considered present when an inoculation loop formed a viscous string of 5 mm or more in length from a bacterial colony [11]. Capsular polysaccharide synthesis (CPS) and virulence-associated genes were detected by multiplex PCR as described by Compain et al. [12]. The presence of CPS genes was reconfirmed by uniplex PCR.

### Multilocus sequence typing, allelic diversity, and population structure analysis

Multilocus sequence typing (MLST) was conducted according to the method of Diancourt et al. [13] and sequence types (STs) were assigned through the Institut Pasteur (Paris, France) database (http://bigsdb.pasteur.fr/klebsiella/klebsiella.html). Individual locus sequences, as well as the concatenated sequences of all seven MLST loci for each isolate, were analyzed for their diversity using DIVEIN [14]. Clonal groups were defined based on STs sharing six loci (single-locus variants) using eBURST [15]. A population snapshot was also drawn for the clonal relationship of these STs with those in the database of the Institut Pasteur using eBURST. Using the alignments of the concatenated sequences from the seven MLST loci for all isolates, a phylogenetic tree was constructed by the neighbor-net method using SplitsTree4 [16].

## Results

Here, we present our findings on multidrug/carbapenem-resistant *KP* isolates obtained at a tertiary care hospital in Riyadh between January 2011 and December 2012. The demographic data on these isolates is given in Table 1.

**Table 1:** Showing the distribution of resistance determinants, plasmid replicons and virulence factors in 71 KP isolates

Isolate	ST	Sampling Date	Speciman	Carbapenemase gene	ESBLs	Other β-Lactamase	Plasmid Replicon
RD- 51	37	27-Oct-11	Blood	OXA-48	CTX-M 15	TEM-1, SHV-11	L/M, FIIK
RD- 52	974	22-Oct-11	wound	OXA-48	CTX-M 15	TEM-1, SHV-11	FIIK, FII, L/M
RD- 53	29	8-Aug-11	Rectal	OXA-48	CTX-M 15	TEM-1, SHV-1	FIIK, L/M
RD- 54	37	9-May-11	Abd. Drain	OXA-48	CTX-M 15	TEM-1, SHV-11	L/M, FIIK
RD- 56	37	31-Jul-11	Sputum	OXA-48	CTX-M 15	TEM-1, SHV-11	L/M, FIIK
RD- 57	694	4-Sep-11	Urine	Negative	CTX-M 15	SHV-1	FIIK, L.M
RD- 58	199	9-Sep-11	Wound	Negative	CTX-M 14	TEM-1, SHV-1	L/M
RD- 59	709	5-Sep-11	Blood	OXA-48	ND	SHV-11	L/M
RD- 60	340	28-May-11	Trachea	Negative	CTX-M 15	SHV-11	FII, FIIK
RD- 61	37	24-Sep-11	Wound	OXA-48	CTX-M 15	TEM-1, SHV-11	L/M, FIIK
RD- 62	709	9-Jan-11	Blood	OXA-48	CTX-M 14	SHV-11	L/M
RD- 63	37	28-Jan-11	Sputum	Negative	CTX-M 15	TEM-1, SHV-11	FIIK
RD- 64	15	9-Jan-11	Trachea	Negative	CTX-M 15	SHV-1	FII, L/M, IncN
RD- 66	199	13-Oct-11	Wound	OXA-48	CTX-M 14	TEM-1, SHV-1	L/M
RD- 67	348	7-Feb-11	Rectal	OXA-48	ND	TEM-1, SHV-11	FIIK, L/M
RD- 68	199	27-Jul-11	Rectal	OXA-48	ND	TEM-1, SHV-1	L/M
RD- 69	974	8-Jan-11	Urine	OXA-48	CTX-M 15	TEM-1, SHV-11	FIIK, L/M
RD- 70	48	26-Mar-11	Trachea	OXA-48	CTX-M 15	TEM-1, SHV-1	FIIK
RD- 71	29	10-Jun-11	Wound	OXA-48	CTX-M 15	TEM-1, SHV-1	L/M
RD- 72	37	2-Feb-11	Blood	OXA-48	ND	TEM-1, SHV-11	L/M, FIIK
RD- 73	199	21-Mar-11	Wound	OXA-48	CTX-M 14	TEM-1, SHV-11	L/M, FIIK
RD- 74	111	24-Mar-11	Wound	OXA-48	CTX-M 14	TEM-1, SHV-11	IncN
RD- 75	199	30-Mar-11	Trachea	OXA-48	CTX-M 14	TEM-1, SHV-1	L/M, FIIK, IncN
RD- 76	37	8-Feb-11	Trachea	OXA-48	CTX-M 15	TEM-1, SHV-11	L/M, FIIK
RD- 78	152	14-Oct-11	Rectal	OXA-48 + NDM-1	CTX-M 15	TEM-1, SHV-1	Negative
RD- 79	37	31-Oct-11	Trachea	OXA-48	CTX-M 15	TEM-1, SHV-11	L/M, FIIK
RD- 80	199	13-Nov-11	Wound	OXA-48	CTX-M 14	TEM-1, SHV-1	L/M, FIIK
RD- 82	37	24-Nov-11	Trachea	OXA-48	ND	TEM-1, SHV-11	L/M, FIIK
RD- 83	348	28-Nov-11	Rectal	OXA-48 + NDM-1	CTX-M 15	TEM-1, SHV-1	FIIK, L/M
RD- 84	340	3-May-12	Trachea	Negative	CTX-M 15	SHV-11	FII, FIB, FIA, FIIK
RD- 85	340	9-Nov-12	Urine	OXA-48	CTX-M 15	SHV-11	FIIK
RD- 86	152	16-Feb-12	Urine	NDM-1	CTX-M 15	TEM-1, SHV-1	FIA
RD- 87	17	6-Nov-12	Trachea	OXA-48	CTX-M 15	SHV-11	FIIK, L/M
RD- 88	152	3-Jan-12	Wound	NDM-1	CTX-M 15	TEM-1, SHV-1	FIB
RD- 89	298	6-Oct-12	Urine	OXA-48	CTX-M 15	TEM-1, SHV-1	FIIK, L/M, IncN
RD- 90	37	6-May-12	Blood	OXA-48	ND	TEM-1, SHV-11	L/M, FIIK
RD- 92	199	22-Jul-12	Rectal	OXA-48	CTX-M 14	TEM-1, SHV-1	L/M, FIIK
RD- 93	199	22-Jan-12	Trachea	OXA-48	CTX-M 14	TEM-1, SHV-1	L/M, FIIK, IncN
RD- 94	152	15-Apr-12	Trachea	NDM-1	CTX-M 15	TEM-1, SHV-1	FIB, FIA
RD- 95	340	16-Mar-12	Blood	Negative	CTX-M 15	SHV 11	FIIK
RD- 96	16	16-Jul-12	Trachea	OXA-48	CTX-M 15	TEM-1, SHV-1	FII, FIIK, FIA
RD- 97	48	1-Dec-12	Trachea	OXA-48	CTX-M 14	TEM-1, SHV-1	FIIK
RD- 99	48	12-Jan-12	Rectal	OXA-48	CTX-M 15	TEM-1, SHV-11	FIB, FIIK, L/M
RD- 100	37	2-Jul-12	Urine	OXA-48	CTX-M 15	TEM-1, SHV-11	L/M, FIIK
RD- 102	37	1-Sep-12	TIP	OXA-48	CTX-M 15	TEM-1, SHV-11	L/M, FIIK
RD- 103	199	7-May-12	Rectal	OXA-48	CTX-M 15	TEM-1, SHV-1	L/M, FIIK
RD- 104	37	25-Jul-12	Wound	OXA-48	CTX-M 15	TEM-1, SHV-11	L/M, FIIK
RD- 105	152	29-Jan-12	Blood	OXA-48 + NDM-1	CTX-M 15	TEM-1, SHV-1	L/M, IncN
RD- 106	152	27-Jul-12	Blood	NDM-1	CTX-M 15	TEM-1, SHV-1	IncN
RD- 107	29	22-Oct-12	Trachea	OXA-48	CTX-M 15	TEM-1, SHV-1	FIIK, L/M, IncN
RD- 108	199	14-Aug-12	Wound	OXA-48 + NDM-1	CTX-M 15	TEM-1, SHV-1	L/M, FIIK
RD- 109	199	16-Feb-12	Urine	OXA-48	CTX-M 14	TEM-1, SHV-1	L/M, FIIK
RD- 111	152	26-Mar-12	Urine	NDM-1	CTX-M 15	TEM-1, SHV-1	Negative
RD- 112	15	28-Oct-12	Wound	OXA-48	CTX-M 15	SHV-1	FIIK, L/M
RD- 113	199	4-Oct-12	Trachea	OXA-48	CTX-M 14	TEM-1, SHV-1	L/M, FIIK
RD- 114	16	18-Nov-12	Trachea	OXA-48	CTX-M 15	TEM-1, SHV-1	FII, FIIK, FIA
RD- 116	48	7-Jun-12	Rectal	OXA-48	CTX-M 15	TEM-1, SHV-11	FIIK
RD- 117	16	8-Jun-12	Urine	OXA-48	CTX-M 15	TEM-1, SHV-1	FII, FIIK, L/M, FIA
RD- 118	152	7-Aug-12	Trachea	OXA-48 + NDM-1	CTX-M 15	TEM-1, SHV-1	L/M, FIB
RD- 119	709	3-Dec-12	Wound	OXA-48	CTX-M 14	SHV-11	L/M
RD- 120	48	11-Mar-12	Rectal	OXA-48	CTX-M 15	TEM-1, SHV-1	FIB, FIA
RD- 121	15	6-Nov-12	Rectal	Negative	CTX-M 15	TEM-1, SHV-1	FIIK
RD- 122	11	17-Nov-12	Urine	OXA-48	CTX-M 15	TEM-1, SHV-11	FIIK, IncI1, L/M
RD- 123	353	17-Nov-12	Rectal	OXA-48	CTX-M 15	TEM-1, SHV-11	L/M, FIIK
RD- 124	152	17-Sep-12	Urine	OXA-48 + NDM-1	CTX-M 15	TEM-1, SHV-1	Negative
RD- 125	199	11-Aug-12	Wound	OXA-48	CTX-M 14	TEM-1, SHV-1	L/M, FIIK, IncN
RD- 126	199	25-Jun-12	Trachea	OXA-48	CTX-M 14	TEM-1, SHV-1	L/M, FIIK
RD- 127	152	12-May-12	Trachea	NDM-1	ND	TEM-1, SHV-1	Negative
RD- 128	1045	12-Nov-12	Urine	NDM-1	ND	TEM-1, SHV-11	FIIK
RD- 129	152	14-Dec-12	Urine	NDM-1	CTX-M 15	TEM-1, SHV-1	FIB
RD- 130	152	25-Nov-12	Rectal	NDM-1	ND	TEM-1, SHV-1	FIB, FIA, IncN

### Isolates and their minimum inhibitory concentrations

Results on resistance determinants, virulence factors, plasmid types, and clonality are presented in Table 1. The clinical sources of the specimen were as follows; respiratory (*n* = 24); surgical wound (*n* = 14); rectal swabs (*n* = 14); urine (*n* = 13); blood (*n* = 9); and abdominal drainage (*n* = 2). Isolates RD-121 and RD-122, which were retrieved from different sites (rectal swab and urine sample) of a single patient 6 months apart, belonged to different STs (STs-15 and -11, respectively) and were considered independent isolates. All isolates were resistant to more than three types of antibiotic. Isolates with ST-199 were all resistant to amikacin (15/15), ‘intermediate’ to tobramycin (9/11), and susceptible to gentamicin (14/15). In contrast, isolates with ST-16 tended to be more susceptible to amikacin and gentamicin than tobramycin. ST-340 isolates were susceptible to amikacin alone. MIC levels are shown in Additional file 1.

### Genes of resistance and their clonal distributions

The *OXA-48* gene was the most common carbapenemase gene, found in 54/71 isolates, followed by the metallo-β-lactamase gene *NDM-1*. Of the 54 isolates positive for *OXA-48*, 48 exhibited *OXA-48* alone and six exhibited *OXA-48* in combination with *NDM-1*. Sequence analysis of the ~ 740-base pair PCR product revealed an *OXA-48* gene allele sensu stricto in all the positive isolates. *OXA-48* was found in the majority of the clones in this study; however, it was less prevalent in STs-152, − 340, and − 15. Among them, four of 12 isolates of ST-152, none of five isolates of ST-340, and one of three isolates of ST-15 were positive for this *OXA-48* gene. The gene *NDM-1* was less common, existing as the lone carbapenemase in nine isolates and in combination with *OXA-48* in six isolates. The main clone carrying this gene was ST-152, with all of its 12 isolates positive for *NDM-1*. No other allelic form of this gene was detected in any isolate. The ~ 1-k base amplicon generated to study the genetic environment surrounding *NDM-1* revealed the presence of *ISAba125 and ble*_MBL_ flanking this gene. The co-existence of two carbapenemase genes (*NDM-1* + *OXA-48*) was seen in six of 71 (8.5%) isolates. Eight isolates were negative for both the *OXA-48* and *NDM-1* genes. This included three isolates from ST-340, two from ST-15, and one each from STs-37, − 199, and − 694. None of the isolates, including those eight that were negative for the *OXA-48* and *NDM-1* genes, were positive for other carbapenemase genes such as *KPC*, *IMP*, and *VIM*. The *CTX-M-15* gene allele was the major extended-spectrum β-lactamase (ESBL), with gene positivity in 47 isolates (69%), whereas *CTX-M-14* was found in 16 isolates (21.1%). *CTX-M-15* was carried by isolates of all STs except STs-199 and -709, whose main *CTX-M* phenotype was *CTX-M-14*. Eleven of 15 isolates of ST-199 and two of three isolates of ST-709 were carriers of the *CTX-M-14* gene. The other *β-lactamase* genes identified in this collection of isolates were *TEM-1* in 60/71 (84.5%) and *SHV* (*SHV-1* and *SHV-11* together) in 71/71 (100%) isolates. Isolates belonging to STs-340 and -709 were all negative for the *TEM-1* gene. Two of three isolates of ST-15 were also negative for the *TEM-1* gene. Isolates belonging to STs-37, − 340, − 709, and − 974 carried the *SHV-11* phenotype, whereas *SHV-1* was the main allele in the remaining STs (Table 1).

### Plasmid profile

PBRT detected seven plasmid replicon types in the whole population of 71 isolates. The replicons identified were: L/M; IncFII; IncFIIK; IncFIA; IncFIB; IncI1; and IncN. Sixty-seven isolates carried one or more plasmid replicons, whereas four isolates were negative for any plasmid type studied. IncFIIK was the predominant plasmid, found in 49/71 (69%) isolates, followed by L/M, found in 47/71 (66.2%). Similarly, the incidence of IncN was 10/71 (14.1%), that of FIA was eight of 71 (11.3%), that of FIB was eight of 71 (11.3%), that of FII was five of 71 (7%), and that of IncI1 was one of 71 (1.4%). The four isolates negative for any plasmid type belonged to ST-152. RD-122 was the only isolate carrying the IncI1 plasmid replicon (Table 1).

### Virulence genes and hypermucoviscocity phenotyping

The status of different virulence genes is shown in Table 1. All isolates were positive for *entB* and 68/71 (95.7%) isolates were positive for *mrkD*. Isolates belonging to ST-709 alone showed the plasmid borne *iutA* virulence factor. All were negative for *rmpA* and the *allS*, *magA* (*K1*), and *K2* CPS genes. Similarly, none of the isolates showed the hypermucoviscocity phenotype (Table 1).

### Conjugation experiments

The *OXA-48* and *NDM-1* genes from these isolates could be transferred into the recipient J53 Az^r^ *E. coli* strain by the process of conjugation. The transconjugants were screened by colony PCR using gene-specific primers (Table 2). These experiments showed that all *NDM-1*-positive isolates tested (*n* = 3) could transfer the gene to their transconjugants. Similarly, the *OXA-48* gene could be transferred from all positive isolates tested (*n* = 10) except for RD-122, the only isolate belonging to ST-11, which failed to transfer its *OXA-48* gene. In contrast, only the *CTX-M-15* gene could be transferred to the recipients, whereas the *CTX-M-14* gene could not. These experiments also showed that the L/M, FIIK, and FII plasmids are conjugative plasmids and can be transferred into recipient bacteria, whereas the IncN1, IncI1, FIA, and FIB plasmids cannot. The results of these experiments are presented in Table 2.

**Table 2:** Results of conjugation experiments on selected isolates of KP

Isolate	Resistance gene(s)/ Plasmid replicon(s)	
	Clone	Transconjugant
RD-99	CTX-M-15, OXA-48/ L/M, FIIK	CTX-M-15, OXA-48/ L/M, FIIK
RD-76	CTX-M-15, OXA-48/ L/M, FIIK	CTX-M-15, OXA-48/ L/M, FIIK
RD-75	CTX-M-14, OXA-48/ L/M, FIIK, IncN1	OXA-48/ L/M, FIIK
RD-62	CTX-M-14, OXA-48/ L/M	OXA-48/ L/M
RD-123	CTX-M-15, OXA-48/ L/M, FIIK	CTX-M-15, OXA-48/ FIIK
RD-122	CTX-M-15, OXA-48/ L/M, FIIK, IncN1	CTX-M-15, − / FIIK
RD-114	CTX-M-15, OXA-48/ FIIK, FII	CTX-M-15, OXA-48/ FIIK, FII
RD-112	CTX-M-15, OXA-48/ L/M, FIIK	CTX-M-15, OXA-48/ L/M, FIIK
RD-118	CTX-M-15, OXA-48, NDM-1/ L/M, FIB	CTX-M-15, OXA-48, NDM-1/ L/M
RD-127	CTX-M-15, NDM-1/ -	CTX-M-15, NDM-1/ -
RD-129	CTX-M-15, NDM-1/ FIB	CTX-M-15, NDM-1/ -
RD-64	CTX-M-15, OXA-48/ L/M, FII, IncN1	CTX-M-15 / L/M, FII

### Multilocus sequence typing, clonal distribution, and genetic diversity

**Fig. 1:**
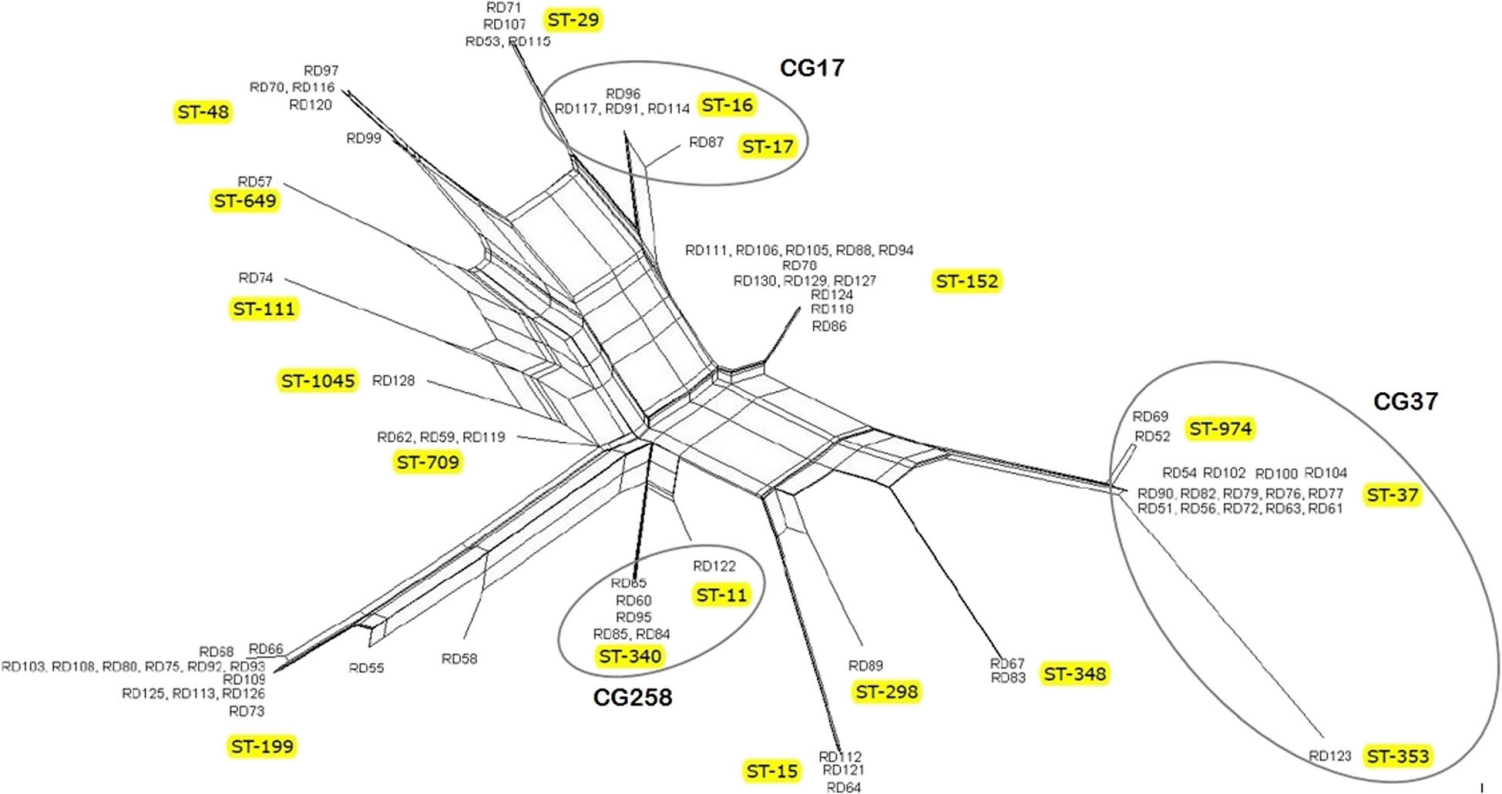
Clonal diversity and phylogenetic relationship among 76 *Klebsiella pneumoniae* isolates. SplitTree4 analysis of the concatenated sequences of the seven loci. The numbers at the tips of the edges represent the isolate identity. Sequence type numbers are indicated beside the isolates/clusters and highlighted

**Fig. 2:**
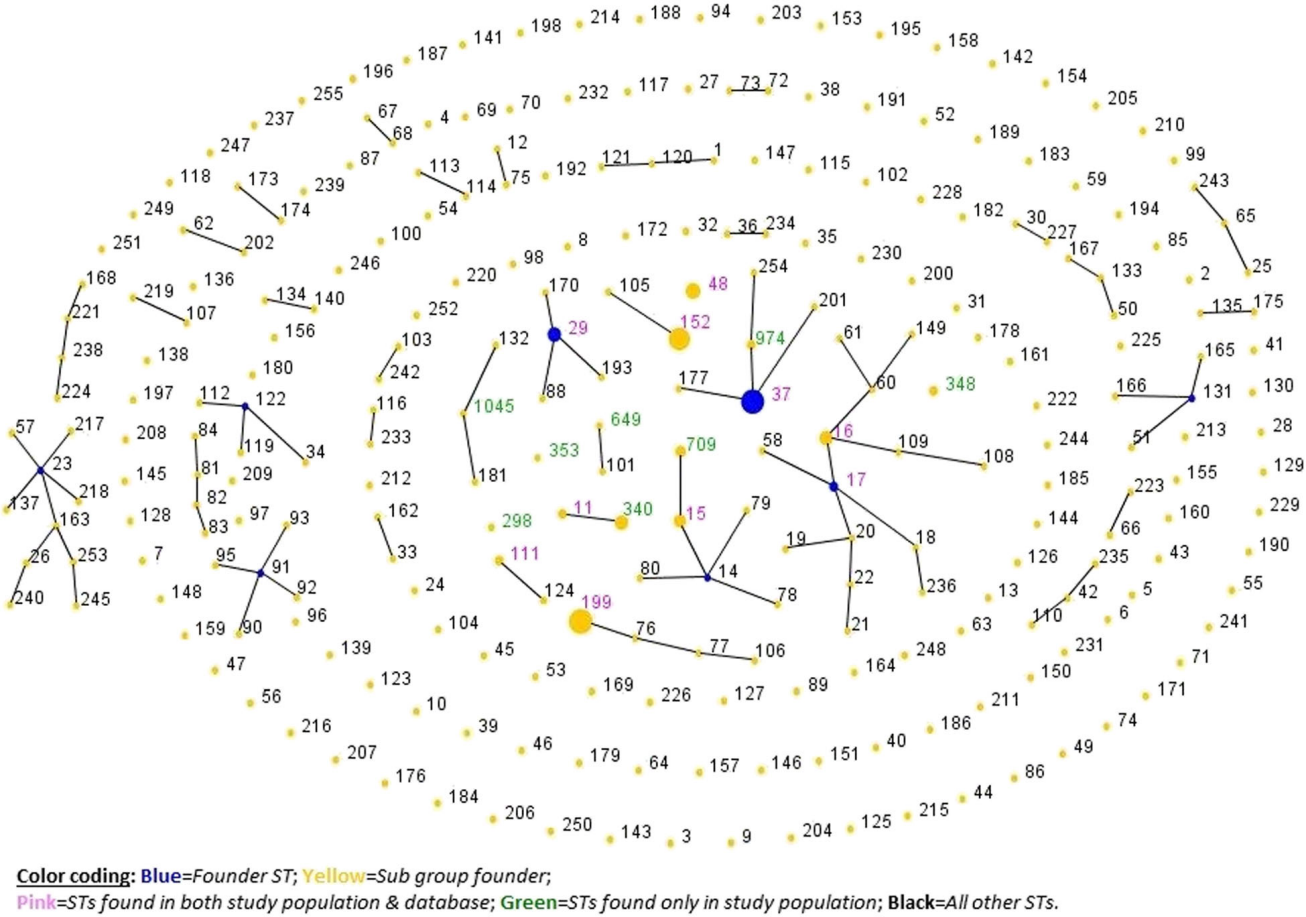
A population snapshot showing the sequence types of the study population of *Klebsiella pneumoniae* isolates compared with the entire multilocus sequence typing database of 3442 isolates of the Institute Pasteur. The size of the circles is proportionate to the number of isolates. For convenience, the study population is placed in the center of the diagram

The 71 isolates sequenced for the seven loci of the MLST scheme of Diancourt et al. [13] exhibited a total of 37 alleles and were distributed into 18 haplotypes/STs. The clonal distribution of the isolates was as follows: ST-199, *n* = 15; ST-152, *n* = 12; ST-37, *n* = 14; ST-48, *n* = 5; ST-340, *n* = 5; ST-29, *n* = 4; ST-16, *n* = 4; ST-709, *n* = 3; ST-15, *n* = 4; ST-348, *n* = 2; ST-974, *n* = 2; and seven other STs with single isolates each. Thirty-four isolates (48%) were distributed in four clonal complexes/groups (CGs) and remained as singletons. A SplitsTree4 diagram was drawn to show their phylogenetic relationship based on the concatenated sequences of all seven loci (Fig. 1). A population snapshot showed their relationship with the STs in the database of the Institut Pasteur (Fig. 2). The 37 alleles ranged from two (*rpoB*)–10 (*tonB*). There were no indels or tri-allelic single-nucleotide polymorphisms (SNPs). Of the 436 SNPs observed, 326 were transitional changes, whereas the remaining 110 were transvertional. The diversity index (π) ranged from 0.00055 (*pgi*)–0.0089 (*tonB*). Of the 39 mutations observed, 31 were synonymous, whereas eight were non-synonymous. Of the eight non-synonymous mutations, six were found at the *tonB* locus. The non-synonymous:synonymous mutations ratio (Ka:Ks) was 0.11 for the concatenated sequences of the seven loci (Table 3). The diversities of the concatenated sequences of the seven loci and individual locus sequences are given in Additional files 2 and 3.

**Table 3:** Nucleotide polymorphism and allelic diversity among the loci of MLST

Locus	Length (bp)	No. alleles present	No. Polymorphic sites (%)	No. Synonymous mutations	No. Non-synonymous mutations	# SNPs	Ts	Tv	Ks	Ka	Ka/Ks	Diversity Index (π)
*gapA*	450	4	4 (0.89)	4	0	32	26	6	0.0060	NAN^a^	–	0.00161126
*InfB*	318	5	4 (1.25)	4	0	69	69	0	0.0160	NAN	–	0.00393538
*mdh*	477	5	7 (1.47)	6	1	35	33	2	0.0050	0.0000	0.0000	0.0014328
*pgi*	432	3	2 (0.46)	2	0	9	0	9	0.0020	NAN	–	0.00055167
*phoE*	420	8	8 (1.90)	8	0	77	73	4	0.0230	NAN	–	0.00533477
*rpoB*	501	2	1 (0.20)	0	1	22	22	0	NAN	0.0010	–	0.00083368
*tonB*	414	10	13 (3.14)	7	6	192	103	89	0.0160	0.0060	0.3750	0.0089006
Concatenated	3012	0	39 (1.29)	31	8	436	326	110	0.0090	0.0010	0.1100	0.00304244

^a^Not a number; *SNP* single nucleotide polymorphism, *Ts* Transitions, *Tv* Transversions, *Ks* Average number of nucleotide substitution per aynonymous site, *Ka* Average number of nucleotide substitution per non-synonymous site

## Discussion

Although a few reports are available on the antibiotic resistance profiles of CR*KP* and other members of the Enterobacteriaceae family in Saudi Arabia [4, 6, 8, 17], there is a paucity of studies on other characteristics such as the plasmid profiles, virulence factors, and clonality of CR*KP*. To the best of our knowledge, this is the first comprehensive report on these characteristics in multidrug-resistant CR*KP* isolates from a single hospital setting in this country.

### Clonality and resistance profile

Multidrug resistance in members of the Enterobacteriaceae family has become a global phenomenon and for several reasons Saudi Arabia is viewed as a growing pool of these pathogens [4, 18–20]. The presence of large numbers of resistance determinants such as *CTX-M, OXA-48, NDM-1, SHV*, and *TEM-1* in our *KP* isolates is reasonable, because all of them are resistant to several classes of antibiotic (Table 1, Additional file 1). A polyclonal *OXA-48* gene was the most common carbapenemase found in our isolates and was distributed among all the STs. The *OXA-48* gene has been reported to be endemic in Saudi Arabia [4, 8]. Although variants of *OXA-48* such as *OXA-204*, − *232*, − *181*, − *162,* and − *163* have also been reported in this region [4, 17, 21], none of our isolates showed any other *OXA-48* variant. Similarly, the incidence of infections involving the *NDM-1* gene is also being regularly reported in Asian countries, but limited data are available on the characteristics of this newly described gene in this region [4]. More recently, Mantilla-Calderon et al. [22] found the *NDM-1* gene on an IncF plasmid in *E. coli* present in a non-nosocomial sample (sewage water) collected in Jeddah, Saudi Arabia, in 2013. The gene appeared to be on a conjugative plasmid because all of their *E. coli* transconjugants were positive for *NDM-1*. However, the genetic environment of *NDM-1* on plasmids carrying the gene in our isolates appeared similar to that reported previously in *KP* and most other *NDM-1* producers of the *Enterobacteriaceae family:* surrounded upstream by an *ISAba125* insertion element and downstream by the bleomycin resistance gene *ble*_MBL_ [23, 24]. Regarding the eight isolates that did not present any of the carbapenemase genes and yet were resistant to carbapenem, an alternative mechanism such as a defective or non-functional outer membrane porin, frequently reported in CR*KP*, may be present [25–27]. The *CTX-M* gene is associated with antibiotic resistance worldwide and its positivity rate of 93% (66/71) in our isolates indicates its endemicity in this region, in agreement with previous reports [8, 28–30]. The presence of *CTX-M-15* (77%) and *CTX-M-14* (23%) variants is also in agreement with previous reports from Saudi Arabia [31]. The conjugation experiment showed that *CTX-M-15*, *OXA-48*, and *NDM-1* genes can be transferred to recipient *E. coli,* whereas *CTX-M-14* appears to be on a non-conjugative plasmid, also evident in its limited dissemination.

### Plasmid typing

Limited data are available on plasmid replicon typing in multidrug-resistant *KP* in Saudi Arabia. The incidence of seven types of plasmid replicon, ranging from one to four in a single isolate, is reasonable in the context of the large number of antibiotic-resistance determinants found in these isolates. The high incidence of conjugative FIIK (69%) and L/M plasmids (66%) found in our isolates is in agreement with reports from European and Asian countries [29, 32–34]. IncI1 is carried only by an isolate with ST-11, a member of clonal group 258, which is famous for carrying the *KP*C gene worldwide. The reason that four isolates lack any plasmid type may be due to differences in their geographic distribution or limitations in the PBRT protocol for their detection [35]. Except for IncI1, FIA, and FIB, all other plasmids could be transferred to recipient *E. coli* during conjugation, indicating that these plasmids contribute to the multidrug-resistant phenotype of these isolates. Because the conjugation experiments were carried out on a limited number of isolates in this study (Table 2), it is not possible to determine the relationship between the replicons identified and resistance determinants. However, these data may provide the international community with information on local plasmid profiles and their evolutionary origins [35].

### Virulence factors

Hypervirulence is usually linked to the presence of the *rmpA*, *magA* (K1), and *K2* genes and the hypermucoviscous phenotype is regarded as critical for an isolate to be virulent, whereas other virulence factors appear to be only contributory to this property [36]. Because all our isolates were negative for the *rmpA*, *magA* (K1), and *K2* genes and did not present a hypermucoviscous phenotype (Table 1), they may have not been hypervirulent-*KP*. These results also support the notion that virulence and antibiotic resistance are independent and not overlapping traits of *KP* [37]. Although none of our isolates showed a hypervirulent phenotype, the presence of siderophores (such as *ybtS*, *entB,* and *iutA*) may contribute to the severity of infections in patients, as reported previously [38]. The virulence factor *iutA* and hypermucoviscous phenotype gene *rmpA* are usually carried together and co-transferred simultaneously by the plasmid pLVPK and its homologs; thus, the presence of *iutA* alone in ST-709 isolates warrants further investigation.

### Clonality and genetic diversity

MLST is a valuable tool for drawing inferences on genetic diversity and population structure in an epidemiologic setting (https://pubmlst.org/references.shtml) [39]. A significant observation of this study is the presence of a polyclonal pool of several genetically unrelated and non-popular antibiotic-resistant *KP* STs at our hospital. Using the Breurec et al.[40]. Approach of grouping of single-and double-locus variants into a clonal group and applying e-BURST analysis, *3*5/71 (49%) isolates (belonging to 10 STs) were grouped in three clonal groups (CG-37, CG258, and CG17), whereas 51% remained singletons belonging to eight diverse and unrelated non-epidemic genetic lineage STs (Fig. 1). A population snapshot drawn against the *KP* MLST database of the Institut Pasteur also showed that only four of 18 (22%) STs aligned with any of known clonal group (Fig. 2). These findings are consistent with those of previous reports that described the spread of ESBL-producing *KP* largely as multi-clonal, in contrast to the spread of KPC-producing *KP*, which is limited to specific clones (CG258) [41]. None of our isolates, including those from CG258, carried a *KPC* gene. Several clones in this study, such as STs-29, − 37, − 709, and − 111, have been previously reported at this hospital as well as elsewhere [8, 42, 43]. These results also support previous observations that this region is becoming a pool for new antibiotic-resistant *KP*, but is still mostly free of *KP*C [4]. The most interesting finding is the presence in large numbers of the non-popular, singleton clone ST-199, which requires further investigation.

Nucleotide diversity in the seven loci sequences remained much lower and the Ka:Ks ratio of the concatenated sequences of these loci was 0.11, indicating a purifying selection against deleterious mutations in these isolates (Table 3). The diversity analysis of individual locus sequences showed a significant diversity in three loci: viz., *infB*, *rpoB,* and *tonB*. Of these, the *tonB* gene appears to be under more selection pressure because six of its 13 polymorphic sites showed non-synonymous mutations (Table 3). A clone-wise analysis of these sequences showed significant diversity between the *tonB* and *infB* genes of the ST-199 and -48 isolates (Fig. 1, Additional file 2). The maximum number of non-synonymous mutations in *tonB* in a single ST (three of six) belonged to ST-199, indicating strong positive selection of this clone.

A significant observation of our study is the association of certain resistance genes with certain clonal types/genotypes of *KP*. For example, the majority of isolates belonging to ST-152 were positive for the *NDM-1* gene and negative for the *OXA-48* gene, whereas isolates of STs-199 and -37 were positive for *OXA-48* and negative for *NDM-1*. Although the *NDM-1* gene is regularly reported [4, 17], *OXA-48* remains the main carbapenemase in this country. We also found this gene in only 21% of the isolates. The interesting feature of this gene was that ST-152 was its main carrier and that all 12 isolates belonging to ST-152 were positive for it. Both this finding and a previous observation from the United Arab Emirates [18] indicate that clone ST-152 of *KP* is a “frequent carrier” of the *NDM-1* gene exclusive to Saudi Arabia. *What makes this more interesting is that all of our isolates belonging to clonal group ST-152 were of the KP subspecies ozaenae, a species not represented in large numbers among KP members* [44]*. This is the first report to describe the involvement of the ozaenae subspecies of KP in the carriage of the NDM-1 gene.* The production of the *OXA-48* and *NDM-1* carbapenemases alone or in combination with other resistance genes, in isolates of genetic lineages that have not previously been described as high-risk clones, is alarming. It suggests that these clones in an outbreak-like situation may limit the spread of other high-risk clones and/or add to their increasing numbers. The *CTX-M-15* phenotype has previously been associated with STs-11, − 15, − 23, and − 37 [45, 46]. In our study population, it was uniformly distributed among all STs except for STs-199 and -709. Isolates from these two clonal types exclusively exhibited *CTX-M-14*. To the best of our knowledge, this association of the *CTX-M-14* phenotype with any ST has not been previously reported. Similarly, the majority of isolates of STs-199 and -709 were *CTX-M-14* carriers. Another example of such an association is that of the *SHV* gene: most of the isolates belonging to STs-199 and -48 were *SHV-11* carriers, whereas *SHV-1* was the primary SHV phenotype in the other clonal groups. Similar selective resistance was observed in STs-199, − 340, and − 16 for different types of aminoglycoside, such as amikacin, gentamycin, and tobramycin.

The incidences of resistance gene profiles among the isolates of different STs indicate that there is a correlation between antibiotic-resistance pattern and clonal group. This is not due to mere clonal expansion of successful clones with certain resistance determinants (Table 4 and Additional files 2 and 3). A correlation between resistance phenotype and the genotypes of bacterial isolates has been reported previously [47]. Similarly, an association between Class-1 integrons, the carriers of resistance genes in bacteria, and certain STs of *KP* has also been reported [48, 49]. However, further studies are necessary to shed light on the mechanism underlying and factors associated with such resistance development in clones that are genetically fit in a particular clinical environment [47]. Our results also suggest a need to explore the mechanism underlying the mutual exclusion of genetic determinants in these phylogenetic groups of *KP* [50].

**Table 4:** eBURST analysis of the MLST data showing clonal groups based on STs and their allelic profiles

Sequence Type	n (% of total)	Clonal Group	Allelic Profile
37	14 (18.4%)	CG37	2–9–2-1-13–1-16
974	2 (2.6%)		4–9–2-1-13–1-16
353	1(1.3%)		3–9–47-1-13–1-16
340	5 (6.6%)	CG258	3–3–1-1-1-1-18
11	1(1.3%)		3–3–1-1-1-1-4
16	4 (5.3%)	CG17	2–1–2-1-4-4-4
17	1(1.3%)		2–1–1-1-4-4-4
15	3 (3.9%)	CG14	1–1–1-1-1-1-1
709	3 (3.9%)		1–1–1-1-1-1-4
348	2 (2.6%)	Singleton	2–1–20-1-12-1-16
298	1(1.3%)	Singleton	4–1–2-1-1-1-7
111	1(1.3%)	Singleton	4–1–5-1-17-1-42
1045	1(1.3%)	Singleton	2–1–1-1-1-1-42
649	1(1.3%)	Singleton	2–1–1-5-4-1-1
199	15 (19.5%)	Singleton	4–34–1-1-21–1-35
152	12 (15.8%)	Singleton	2–3–2-1-1-4-56
48	5 (6.6%)	Singleton	2–5–2-2-7-1-10
29	4 (5.3%)	Singleton	2–3–2-2-6-4-4

## Additional files

Additional file 1: Minimum inhibitory concentrations table. The minimum inhibitory concentrations for various antibiotics seen in 71 *Klebsiella pneumoniae* isolates. (DOCX 22 kb)

Additional file 2: Clonal diversity analysis. Genetic diversity analysis at the nucleotide level across the seven multilocus sequence typing loci concatenated sequences, showing significant diversity for sequence types-48 and -199. (PDF 311 kb)

Additional file 3: Individual diversity graphs. Diversity graphs for each of the seven multilocus sequence typing loci sequences i.e., *gapA*, *mdh*, *pgi*, *phoE*, *rpoB*, *infB*, and *tonB. (PNG 92 kb)*

## Abbreviations

CGs: Clonal complexes/groups
CPS: capsular polysaccharide synthesis
CRKP: Carbapenem-resistant *Klebsiella pneumoniae*
ESBL: Extended-spectrum β-Lactamase
Ka:Ks: Asynonymous:synonymous mutations
KAIMRC: King Abdullah International Medical Research Center
KP: *Klebsiella pneumoniae*
KPC: *Klebsiella pneumoniae* carbapenemase
LB: Luria bertani
MICs: Minimum inhibitory concentrations
MLST: Multilocus sequence typing
PBRT: PCR-based replicon typing
PCR: Polymerase chain reaction
SNPs: Single-nucleotide polymorphisms
STs: Sequence types
