# Supplementary material for: Clonal diversity and genetic profiling of antibiotic resistance among multidrug/carbapenem-resistant Klebsiella pneumoniae isolates from a tertiary care hospital in Saudi Arabia
Source: BMC Infect Dis. 2018 May 3;18:205. doi: 10.1186/s12879-018-3114-9 (PMC5934806; doi:10.1186/s12879-018-3114-9)
Supplement: Supplementary file 1 — Minimum inhibitory concentrations table. The minimum inhibitory concentrations for various antibiotics seen in 71 Klebsiella pneumoniae isolates. (DOCX 22 kb) [file 12879_2018_3114_MOESM1_ESM.docx]

Additional Table: Showing MICS for the various antibiotics seen in 71 KP Isolates.

| Isolate # | Pip/Taz | Amik | Gent | Tobra | Cefepi | Imipe | Merop | Ciproflo | TSX^*^ |
| --- | --- | --- | --- | --- | --- | --- | --- | --- | --- |
| RD- 51 | R (128) | R (64) | R(16) | R(16) | R (64) | R(16) | ND | R (4) | R (320) |
| RD- 52 | ND | R (64) | R(16) | ND | R (64) | R(16) | R (16) | R (4) | R (320) |
| RD- 53 | R (128) | S (2) | R(16) | R(16) | R (64) | R(16) | R (32) | R (4) | R (320) |
| RD- 54 | R (128) | ND | S (1) | ND | R (64) | R (32) | R (32) | ND | ND |
| RD- 56 | R (128) | R (64) | R(16) | R(16) | R (64) | R(16) | R(16) | R (4) | R (320) |
| RD- 57 | R (128) | S (2) | R(16) | ND | R (64) | R(16) | R (32) | R (4) | R (320) |
| RD- 58 | R (128) | R (64) | S (1) | 1(I) | R (64) | R(16) | R(16) | R (4) | R (320) |
| RD- 59 | R | R (64) | R(16) | ND | R (64) | R (8) | R (8) | R (4) | S (20) |
| RD- 60 | ND | S (2) | R(16) | ND | R (64) | I (4) | R(16) | R (4) | ND |
| RD- 61 | R (128) | R (64) | R(16) | R(16) | ND | R(16) | R(16) | R (4) | R (320) |
| RD- 62 | ND | I (8) | I (8) | ND | R (64) | R (4) | R (4) | R (4) | S (20) |
| RD- 63 | ND | S (2) | R(16) | ND | R (64) | R (32) | R(16) | R (4) | R (320) |
| RD- 64 | R (128) | R (64) | R(16) | I (8) | ND | I (4) | R(8) | S | R (320) |
| RD- 66 | R (128) | R (64) | S (1) | I (1) | R (64) | ND | I (1) | R (4) | R (320) |
| RD- 67 | R | R (64) | R(16) | ND | R (64) | I (4) | R(8) | R (4) | R (320) |
| RD- 68 | R (128) | R (64) | R(16) | ND | R (64) | R(16) | R(16) | R (4) | R (320) |
| RD- 69 | ND | R (64) | R(16) | ND | R (64) | S | R (16) | R (4) | R (320) |
| RD- 70 | ND | R (64) | R(16) | ND | R (64) | R (8) | R (16) | R (4) | R (320) |
| RD- 71 | ND | R (64) | R (S) | ND | ND | S (I) | R (32) | R (4) | R (320) |
| RD- 72 | ND | R (64) | R(16) | ND | ND | R (32) | R (1.5) | R (4) | R (320) |
| RD- 73 | R (128) | R (64) | S (1) | ND | R (64) | R (32) | R (32) | R (4) | ND |
| RD- 74 | R (128) | R (64) | R (S) | ND | ND | S (I) | R(16) | R (4) | S (20) |
| RD- 75 | R (128) | R (64) | S (1) | ND | ND | R (32) | R (32) | R (4) | R (320) |
| RD- 76 | R (128) | R (64) | R(16) | ND | R (64) | R (32) | R (32) | R (4) | R (320) |
| RD- 78 | R (128) | R (64) | R (16) | R (16) | ND | R (16) | R (8) | R (4) | R (320) |
| RD- 79 | R (128) | R (64) | R(16) | R(16) | ND | ND | R (8) | R (4) | R (320) |
| RD- 80 | R(128) | R (64) | S (1) | I( 1) | ND | R (64) | R (16) | R (4) | R (320) |
| RD- 82 | R (128) | R (64) | S (1) | S (1) | I (16) | R (16) | R(16) | R (4) | R (320) |
| RD- 83 | ND | I (16) | S (1) | I (8) | R (64) | R (8) | R (16) | S (1) | R (320) |
| RD- 84 | ND | I (16) | R (16) | R (16) | R (64) | R (8) | R (16) | R (4) | R (320) |
| RD- 85 | ND | S (2) | S (1) | S (1) | R (64) | R (8) | R (16) | R (4) | S (20) |
| RD- 86 | R (128) | R (64) | R (16) | R (16) | I (16) | R(4) | R (8) | R (4) | R (320) |
| RD- 87 | R (128) | S (2) | R (16) | R (16) | R (64) | R (1) | R (16) | R (2) | R (320) |
| RD- 88 | R (128) | R (64) | R (16) | R (16) | R (64) | R (4) | R (4) | R (4) | R (320) |
| RD- 89 | R (128) | S 4 | R (16) | R (16) | I(16) | R (4) | R (16) | R (4) | R (320) |
| RD- 90 | ND | R (64) | R(16) | ND | I (2) | R (32) | R (32) | R (4) | R (320) |
| RD- 92 | R (128) | R (64) | S (1) | I( 1) | I(16) | I (2) | I (1) | R (4) | R (320) |
| RD- 93 | R (128) | R (64) | S (1) | I( 1) | R (32) | R (8) | I (1) | R (4) | R (320) |
| RD- 94 | R (128) | R (64) | R (16) | R (16) | ND | R (8) | R (8) | R (4) | S (20) |
| RD- 95 | ND | I (8) | R (16) | R (16) | R (64) | R (1) | I (2) | R (4) | R (320) |
| RD- 96 | R (128) | I 16 | S (1) | R (16) | I (16) | R (4) | R (4) | R (4) | R (320) |
| RD- 97 | R (128) | I 16 | R (16) | R (16) | I (16) | I (2) | I (1) | R (2) | R (320) |
| RD- 99 | ND | I (16) | R (16) | ND | R (64) | R (16) | R (16) | R (4) | R (320) |
| RD- 100 | R (128) | R (64) | R (16) | R (16) | R (64) | R (16) | R (16) | R (4) | R (320) |
| RD- 102 | ND | R (64) | R (16) | ND | R (64) | R (16) | R (32) | R (4) | R (320) |
| RD- 103 | R (128) | R (64) | S (1) | I( 1) | I (8) | R (16) | R (16) | R (4) | R (320) |
| RD- 104 | R (128) | R (64) | R (16) | R (16) | ND | R (16) | R (16) | R (4) | R (320) |
| RD- 105 | R (128) | R (64) | R (16) | R (16) | R (64) | R (16) | R (8) | R (4) | R (320) |
| RD- 106 | R (128) | R (64) | R (16) | R (16) | ND | R (16) | R (4) | R (4) | R (320) |
| RD- 107 | R (128) | S (6) | R (16) | R (16) | R (64) | R (16) | R (16) | R (4) | R (320) |
| RD- 108 | R (128) | I (32) | S (1) | R (16) | R (64) | R (16) | R (16) | R (4) | R (80) |
| RD- 109 | R (128) | R (64) | S (1) | I( 1) | R (64) | R (16) | R (16) | R (4) | R (320) |
| RD- 111 | R (128) | R (64) | R (16) | R (16) | R (64) | R (8) | R (8) | R (4) | R (320) |
| RD- 112 | R (128) | R (64) | S (1) | R (16) | R (64) | R (16) | R (16) | R (4) | S (20) |
| RD- 113 | R (128) | R (64) | S (1) | I( 1) | R (64) | I (2) | I (1) | R (4) | R (320) |
| RD- 114 | R (128) | S (2) | S (1) | S (1) | R (64) | R (8) | R (16) | R (4) | R (320) |
| RD- 116 | R (128) | R (64) | R (16) | R (16) | R (64) | R (8) | R (4) | R (4) | R (320) |
| RD- 117 | R (128) | I 16 | S (1) | R (16) | R (64) | R (4) | I (2) | R (4) | R (320) |
| RD- 118 | R (128) | R (64) | R (16) | R (16) | I (16) | R (16) | R (8) | R (4) | R (320) |
| RD- 119 | ND | I 16 | I (8) | R (16) | R (64) | R (8) | R (8) | R (4) | S (20) |
| RD- 120 | R (128) | S (2) | R (16) | R (16) | R (64) | ND | R (16) | R (4) | R (160) |
| RD- 121 | R (128) | R (64) | R (16) | R (16) | R (64) | R (4) | R (8) | R (4) | S (20) |
| RD- 122 | R (128) | R (64) | R (16) | I (8) | R (64) | R (1) | I (2) | R (4) | S (20) |
| RD- 123 | R (128) | S (2) | R (16) | R (16) | R (64) | ND | R (16) | R (4) | R (320) |
| RD- 124 | R (128) | R (64) | R (16) | R (16) | R (64) | ND | R (8) | R (4) | R (320) |
| RD- 125 | R (128) | R (64) | S (1) | I( 1) | R (64) | R (16) | R (16) | R (4) | R (320) |
| RD- 126 | R (128) | R (64) | S (1) | I( 1) | R (64) | R (4) | I (1) | R (4) | R (320) |
| RD- 127 | R (128) | R (64) | R (16) | R (16) | R (64) | ND | R (16) | R (4) | R (320) |
| RD- 128 | R (128) | S (2) | R (16) | R (8) | R (64) | ND | I (2) | S (0.25) | R (320) |
| RD- 129 | R (128) | R (64) | R (16) | R (16) | R (64) | R (16) | R (8) | R (4) | R (320) |
| RD- 130 | R (128) | R (64) | R (16) | R (16) | R (64) | ND | R (8) | R (4) | R (320) |

Pip/Taz = Piperacillin/Tazobactam; Amik = Amikacin; Gent = Gentamicin; Tobra = Tobramycin; Cefepi = Cefepime;

Imipe = Imipenem; Merop = Meropenem; Ciproflo = Ciprofloxacin; TSX = Trimethoprim + Sulfamethoxazole.

R=Resistant; I=Intermediate; S=Sensitive. The values is parentheses are their MICs.
