# Supplementary material for: Clonal diversity and genetic profiling of antibiotic resistance among multidrug/carbapenem-resistant Klebsiella pneumoniae isolates from a tertiary care hospital in Saudi Arabia
Source: BMC Infect Dis. 2018 May 3;18:205. doi: 10.1186/s12879-018-3114-9 (PMC5934806; doi:10.1186/s12879-018-3114-9)

Diversity Graph for loci **gapA, *mdh, pgi, phoE:***

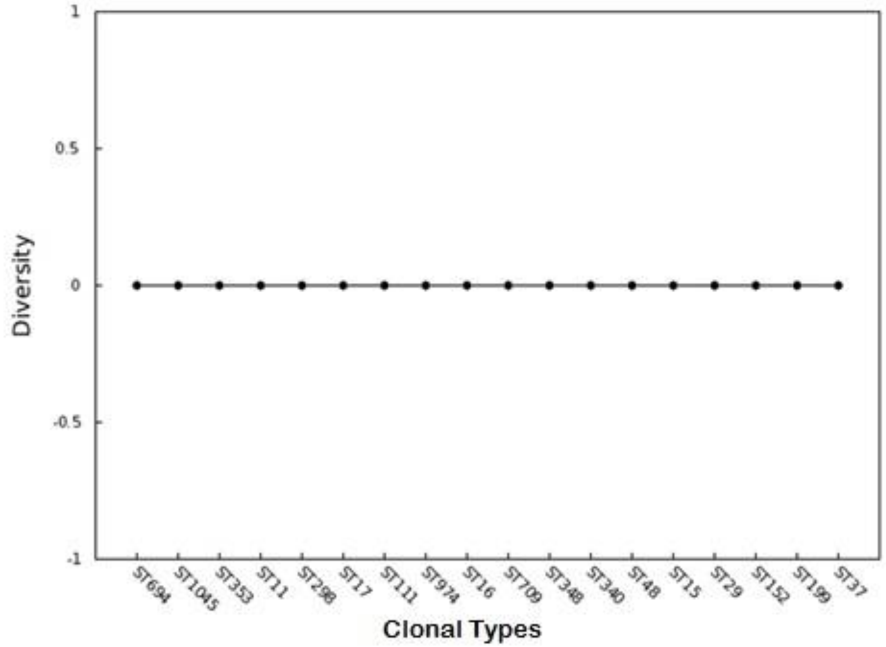

Diversity Graph for locus ***rpoB:***

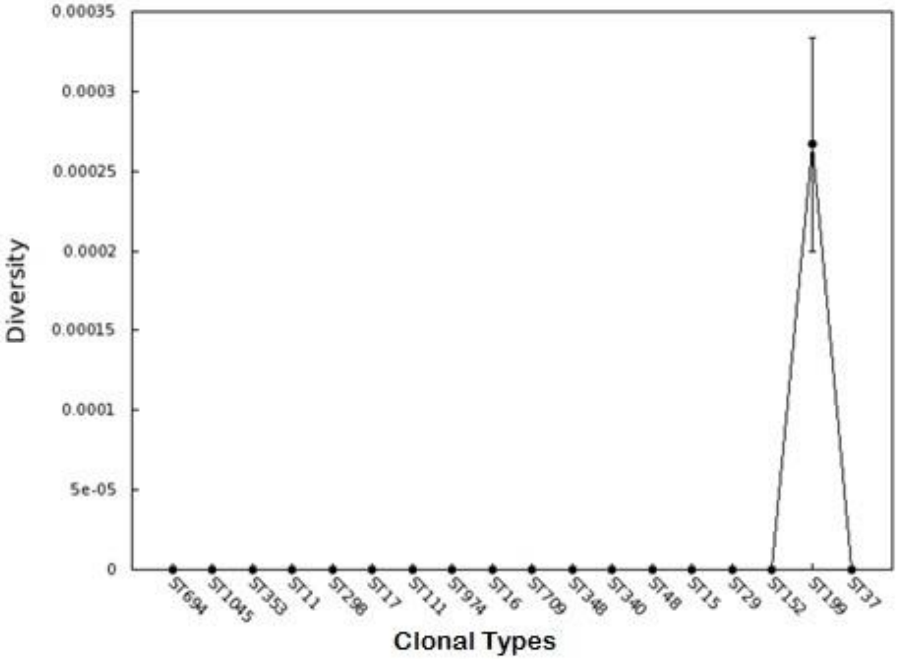

Diversity Graph for locus ***infB:***

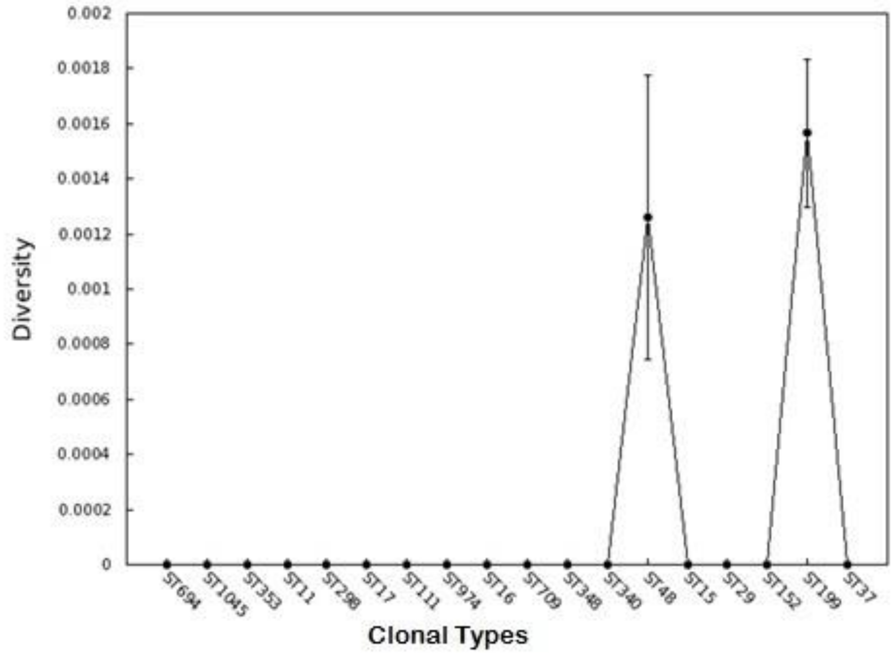

Diversity Graph for locus ***tonB:***

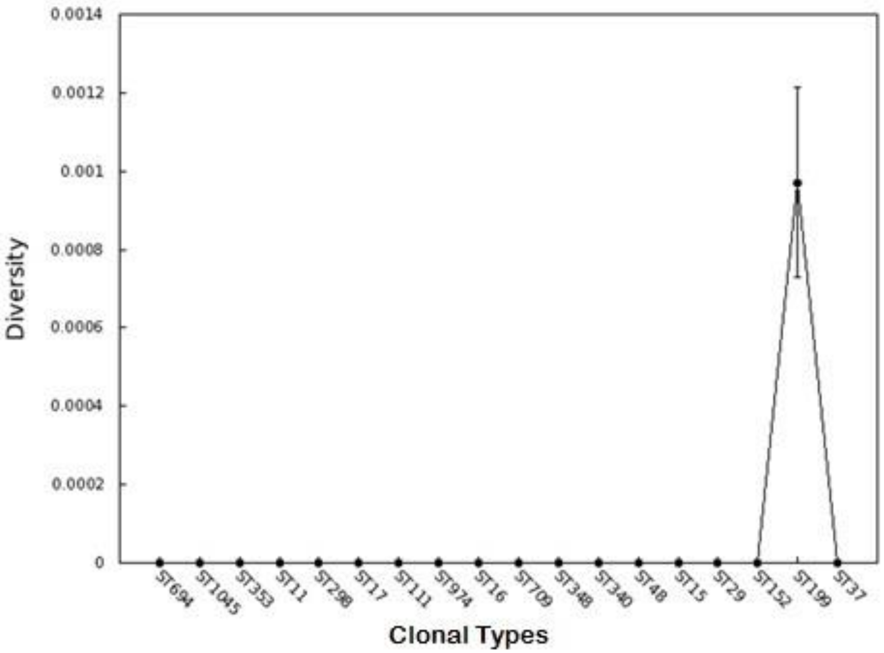

Supplement: Supplementary file 2 — Clonal diversity analysis. Genetic diversity analysis at the nucleotide level across the seven multilocus sequence typing loci concatenated sequences, showing significant diversity for sequence types-48 and -199. (PDF 311 kb) [file 12879_2018_3114_MOESM2_ESM.pdf]
