# Supplementary figures and images for: Clonal diversity and genetic profiling of antibiotic resistance among multidrug/carbapenem-resistant Klebsiella pneumoniae isolates from a tertiary care hospital in Saudi Arabia
Source: BMC Infect Dis. 2018 May 3;18:205. doi: 10.1186/s12879-018-3114-9 (PMC5934806; doi:10.1186/s12879-018-3114-9)

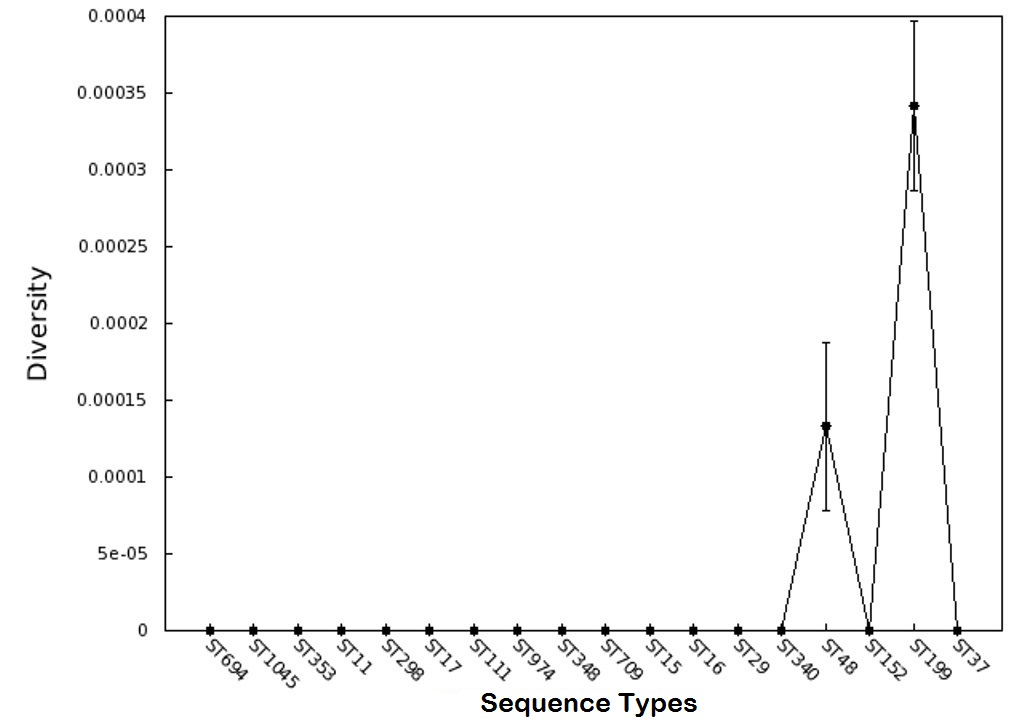

Supplement: Supplementary file 3 — Individual diversity graphs. Diversity graphs for each of the seven multilocus sequence typing loci sequences i.e., gapA, mdh, pgi, phoE, rpoB, infB, and tonB. (PNG 92 kb) [file 12879_2018_3114_MOESM3_ESM.png]
